# Supplementary material for: Association between Dietary Behaviors and BMI Stratified by Sex and the ALDH2 rs671 Polymorphism in Japanese Adults
Source: Nutrients. 2022 Dec 1;14(23):5116. doi: 10.3390/nu14235116 (PMC9741124; doi:10.3390/nu14235116)
Supplement: Supplementary file 1 [file nutrients-14-05116-s001.zip › nutrients-2036965-supplementary.pdf]

Supplementary Materials

Supplementary Table S1. The web-based questionnaire for dietary behaviors in this study

Questions	Answer choices							
How often do you eat meat and fish in a month?								
Beef, pork	I hardly eat.	1 to 3 times a month	1 - 2 days a week	3-4 days a week	5 to 6 days a week	Once a day	Twice daily	More than 3 times daily
Chicken	I hardly eat.	1 to 3 times a month	1 - 2 days a week	3-4 days a week	5 to 6 days a week	Once a day	Twice daily	More than 3 times daily
Lever	I hardly eat.	1 to 3 times a month	1 - 2 days a week	3-4 days a week	5 to 6 days a week	Once a day	Twice daily	More than 3 times daily
Processed meat (ham, sausage, etc.)	I hardly eat.	1 to 3 times a month	1 - 2 days a week	3-4 days a week	5 to 6 days a week	Once a day	Twice daily	More than 3 times daily
Fish (sashimi, boiled fish, grilled fish etc.)	I hardly eat.	1 to 3 times a month	1 - 2 days a week	3-4 days a week	5 to 6 days a week	Once a day	Twice daily	More than 3 times daily
Squid, shrimp, octopus, crab	I hardly eat.	1 to 3 times a month	1 - 2 days a week	3-4 days a week	5 to 6 days a week	Once a day	Twice daily	More than 3 times daily
Shellfish (clams, kaki etc.)	I hardly eat.	1 to 3 times a month	1 - 2 days a week	3-4 days a week	5 to 6 days a week	Once a day	Twice daily	More than 3

								times daily
Processed products (Kamaboko, Chikuwa, etc.)	I hardly eat.	1 to 3 times a month	1 - 2 days a week	3-4 days a week	5 to 6 days a week	Once a day	Twice daily	More than 3 times daily
Fish egg (how much, turtle, etc.)	I hardly eat.	1 to 3 times a month	1 - 2 days a week	3-4 days a week	5 to 6 days a week	Once a day	Twice daily	More than 3 times daily
Small fish to eat with each bone (shishamo, dried shiitake etc.)	I hardly eat.	1 to 3 times a month	1 - 2 days a week	3-4 days a week	5 to 6 days a week	Once a day	Twice daily	More than 3 times daily
Tuna can	I hardly eat.	1 to 3 times a month	1 - 2 days a week	3-4 days a week	5 to 6 days a week	Once a day	Twice daily	More than 3 times daily

How often do you eat eggs, dairy products, oil in a month?

Egg	I hardly eat.	1 to 3 times a month	1 - 2 days a week	3-4 days a week	5 to 6 days a week	Once a day	Twice daily	More than 3 times daily
Milk	I hardly drink it.	1 to 3 months	1 to 2 weeks per week	3-4 per week	5-6 per week	Every day 1	2 or more daily	
Yogurt	I hardly eat.	1 to 3 times a month	1 - 2 days a week	3-4 days a week	5 to 6 days a week	Once a day	Twice daily	More than 3 times daily
Lactic acid bacteria beverage	I hardly drink it.	1 to 3 months	1 to 2 weeks per week	3-4 per week	5-6 per week	Every day 1	2 or more daily	
Butter in the bread	I hardly eat.	1 to 3 times a month	1 - 2 days a week	3-4 days a week	5 to 6 days a week	Once a day	Twice daily	More than 3

								times daily
Margarine to spread in bread	I hardly eat.	1 to 3 times a month	1 - 2 days a week	3-4 days a week	5 to 6 days a week	Once a day	Twice daily	More than 3 times daily
Fried food (fried, tempura, fried etc)	I hardly eat.	1 to 3 times a month	1 - 2 days a week	3-4 days a week	5 to 6 days a week	Once a day	Twice daily	More than 3 times daily
Stir-fry (dish with a small amount of oil)	I hardly eat.	1 to 3 times a month	1 - 2 days a week	3-4 days a week	5 to 6 days a week	Once a day	Twice daily	More than 3 times daily

How often do you eat cereals and potatoes in a month?

Rice	I hardly eat.	1 to 3 times a month	1 - 2 days a week	3-4 days a week	5 to 6 days a week	Once a day	Twice daily	More than 3 times daily
Bread (including confectionery bread)	I hardly eat.	1 to 3 times a month	1 - 2 days a week	3-4 days a week	5 to 6 days a week	Once a day	Twice daily	More than 3 times daily
Noodles	I hardly eat.	1 to 3 times a month	1 - 2 days a week	3-4 days a week	5 to 6 days a week	Once a day	Twice daily	More than 3 times daily
Potato	I hardly eat.	1 to 3 times a month	1 - 2 days a week	3-4 days a week	5 to 6 days a week	Once a day	Twice daily	More than 3 times daily

How often do you eat vegetables, fruits, seaweed etc. in a month?

Yellow vegetables (carrots)	I hardly eat.	1 to 3 times a month	1 - 2 days a week	3-4 days a week	5 to 6 days a week	Once a day	Twice daily	More than 3 times daily

Yellow vegetables (pumpkin)	I hardly eat.	1 to 3 times a month	1 - 2 days a week	3-4 days a week	5 to 6 days a week	Once a day	Twice daily	More than 3 times daily
Green leafy vegetables (spinach, komatsuna etc.)	I hardly eat.	1 to 3 times a month	1 - 2 days a week	3-4 days a week	5 to 6 days a week	Once a day	Twice daily	More than 3 times daily
Green vegetables (broccoli)	I hardly eat.	1 to 3 times a month	1 - 2 days a week	3-4 days a week	5 to 6 days a week	Once a day	Twice daily	More than 3 times daily
Other green-yellow vegetables (green pepper, savory sauce etc.)	I hardly eat.	1 to 3 times a month	1 - 2 days a week	3-4 days a week	5 to 6 days a week	Once a day	Twice daily	More than 3 times daily
Light vegetables (cabbage)	I hardly eat.	1 to 3 times a month	1 - 2 days a week	3-4 days a week	5 to 6 days a week	Once a day	Twice daily	More than 3 times daily
Light vegetables (radish)	I hardly eat.	1 to 3 times a month	1 - 2 days a week	3-4 days a week	5 to 6 days a week	Once a day	Twice daily	More than 3 times daily
Light vegetables (pickles of takuan pickles)	I hardly eat.	1 to 3 times a month	1 - 2 days a week	3-4 days a week	5 to 6 days a week	Once a day	Twice daily	More than 3 times daily
Light vegetables (burdock, bamboo shoots, etc.)	I hardly eat.	1 to 3 times a month	1 - 2 days a week	3-4 days a week	5 to 6 days a week	Once a day	Twice daily	More than 3 times daily
Other pale vegetables (cucumber, onions, bean sprouts, lettuce etc.)	I hardly eat.	1 to 3 times a month	1 - 2 days a week	3-4 days a week	5 to 6 days a week	Once a day	Twice daily	More than 3 times daily

Citrus fruits (oranges, grapefruits, etc.)	I hardly eat.	1 to 3 times a month	1 - 2 days a week	3-4 days a week	5 to 6 days a week	Once a day	Twice daily	More than 3 times daily
Other fruits (strawberries, apples, kiwi etc.)	I hardly eat.	1 to 3 times a month	1 - 2 days a week	3-4 days a week	5 to 6 days a week	Once a day	Twice daily	More than 3 times daily
Seaweed (Hijiki, kelp etc.)	I hardly eat.	1 to 3 times a month	1 - 2 days a week	3-4 days a week	5 to 6 days a week	Once a day	Twice daily	More than 3 times daily
Mushrooms<br>(Shiitake mushrooms, Shimeji, Enoki etc)	I hardly eat.	1 to 3 times a month	1 - 2 days a week	3-4 days a week	5 to 6 days a week	Once a day	Twice daily	More than 3 times daily

How often do you eat or drink confectionary / preference drinks in a month?

Japanese confectionery (Manju etc.)	I hardly eat.	1 to 3 times a month	1 - 2 days a week	3-4 days a week	5 to 6 days a week	Once a day	Twice daily	More than 3 times daily
Pastry (cake, cream puff)	I hardly eat.	1 to 3 times a month	1 - 2 days a week	3-4 days a week	5 to 6 days a week	Once a day	Twice daily	More than 3 times daily
Mayonnaise	I hardly eat.	1 to 3 times a month	1 - 2 days a week	3-4 days a week	5 to 6 days a week	Once a day	Twice daily	More than 3 times daily

How often do you eat bean products a month?

Tofu	I hardly eat.	1 to 3 times a month	1 - 2 days a week	3-4 days a week	5 to 6 days a week	Once a day	Twice daily	More than 3 times daily
Natto, soybeans	I hardly eat.	1 to 3 times a month	1 - 2 days a week	3-4 days a week	5 to 6 days a week	Once a day	Twice daily	More than 3

								times daily
Miso soup	I hardly eat.	1 to 3 times a month	1 - 2 days a week	3-4 days a week	5 to 6 days a week	Once a day	Twice daily	More than 3 times daily
Ganmodoki [a], atsuage dofu [b]	I hardly eat.	1 to 3 times a month	1 - 2 days a week	3-4 days a week	5 to 6 days a week	Once a day	Twice daily	More than 3 times daily
Peanuts, almond								
Coffee (regular, instant)	I hardly drink it.	2 cups or less per week	3-4 cups a week	5 to 6 cups per week	1 - 2 cups daily	3-4 cups daily	More than 5 cups daily	
Coffee (cans, plastic bottles, paper packs)	I hardly drink it.	2 cups or less per week	3-4 cups a week	5 to 6 cups per week	1 - 2 cups daily	3-4 cups daily	More than 5 cups daily	
Japanese green tea (Sencha, Bancha, Gyokuro etc.)	I hardly drink it.	1 - 3 cups daily	4 to 6 cups daily	7 to 9 cups daily	Ten or more cups daily			
Chinese tea (oolong tea, jasmine tea etc.)	I hardly drink it.	2 cups or less per week	3-4 cups a week	5 to 6 cups per week	1 - 2 cups daily	3-4 cups daily	More than 5 cups daily	
Black tea	I hardly drink it.	2 cups or less per week	3-4 cups a week	5 to 6 cups per week	1 - 2 cups daily	3-4 cups daily	More than 5 cups daily	
Vegetable juice (tomato juice etc.)	I hardly drink it.	2 cups or less per week	3-4 cups a week	5 to 6 cups per week	1 - 2 cups daily	3-4 cups daily	More than 5 cups daily	

100% fruit juice	I hardly drink it.	2 cups or less per week	3-4 cups a week	5 to 6 cups per week	1 - 2 cups daily	3-4 cups daily	More than 5 cups daily
Soft drinks, tap water, mineral water etc.	I hardly drink it.	2 cups or less per week	3-4 cups a week	5 to 6 cups per week	1 - 2 cups daily	3-4 cups daily	More than 5 cups daily
Soymilk	I hardly drink it.	1 to 3 months	1 to 2 weeks per week	3-4 per week	5-6 per week	Every day 1	2 or more daily
How often do you drink alcohol?	I don't drink	Everyday	5 to 6 days/week	3 to 4 days/week	1 to 2 days/week	1 to 3 days/month	less than 1 days/month

(Drinkers only) Please tell me the type and amount of alcohol you drink per day ※ Please select the amount to drink or not to drink for all question frames

Japanese Sake （1pair＝180ml）	( ) pair	( ) Times / week
Shochu / Awamori	( ) ml	( ) Times / week
Beer	( ) ml	( ) Times / week
Red wine	( ) ml	( ) Times / week
White wine	( ) ml	( ) Times / week

Chu high / high ball / cocktail	( ) ml	( ) Times / week	
Other alcohol	( )	( ) ml	( ) Times / week
Other alcohol			

[a] Fried tofu mixed with vegetables.

[b] Thick fried tofu

Supplementary Table S2. Characteristics of participants							
		Male				Female		
		Genotype [a]				Genotype [a]		
		GG	GA	AA		GG	GA	AA
Non-alcohol beverage intake, cups/week [b]								
	Regular coffee	8.63 ± 8.97	10.34 ± 9.53	10.49 ± 9.77		9.06 ± 8.51	10.08 ± 9.25	9.80 ± 9.30
	Canned coffee	2.65 ± 5.2	3.08 ± 5.54	3.65 ± 6.68		1.27 ± 3.78	1.34 ± 3.9	1.05 ± 2.93
	Green tea	12.67 ± 13.23	12.73 ± 12.49	13.9 ± 12.89		12.09 ± 12.75	12.51 ± 13.34	14.66 ± 14.99
	Chinese tea	2.26 ± 5.43	2.2 ± 4.97	2.42 ± 5.26		2.21 ± 5.2	2.36 ± 5.44	2.45 ± 5.37
	Black tea	0.83 ± 2.77	1.08 ± 3.2	1.13 ± 2.82		1.66 ± 3.75	1.86 ± 3.98	2.59 ± 4.9
	Milk	1.78 ± 2.57	1.92 ± 2.66	2.03 ± 2.81		1.98 ± 2.65	2.22 ± 2.81	1.99 ± 2.59
	Soy milk	0.43 ± 1.41	0.5 ± 1.59	0.39 ± 1.35		0.83 ± 1.97	0.85 ± 1.97	0.89 ± 2.1
	Lactobacillus beverage	1.18 ± 2.18	1.19 ± 2.14	1.11 ± 2.1		1.08 ± 2.11	1.01 ± 2.02	0.97 ± 1.85
	Vegetable juice	1.72 ± 3.49	1.82 ± 3.6	1.39 ± 2.82		1.34 ± 2.89	1.42 ± 2.94	1.41 ± 3.19
	Fruit juice	0.82 ± 2.1	0.89 ± 2.12	0.93 ± 2.09		0.77 ± 2.1	0.84 ± 2.06	0.89 ± 2.58

	Soft drink	11.27 ± 11.01	10.71 ± 10.67	9.61 ± 10.06		11.51 ± 11.24	11.42 ± 11.31	10.03 ± 10.57
Food intake, times/week [b]								
	Rice	4.90 ± 2.53	5.11 ± 2.46	4.8 ± 2.59		5.03 ± 2.42	5.04 ± 2.42	4.83 ± 2.50
	Bread	2.83 ± 2.69	3.38 ± 2.77	3.86 ± 2.77		3.39 ± 2.81	3.89 ± 2.95	3.97 ± 2.92
	Noodle	2.08 ± 1.76	2.11 ± 1.76	1.96 ± 1.72		1.84 ± 1.67	1.81 ± 1.61	1.80 ± 1.70
	Beaf and pork	4.04 ± 3.39	4.05 ± 3.21	3.93 ± 3.50		4.00 ± 3.14	3.94 ± 3.23	3.86 ± 3.16
	Chicken	2.27 ± 2.10	2.14 ± 1.92	2.02 ± 1.78		2.12 ± 1.79	2.06 ± 1.75	1.89 ± 1.58
	Liver	0.21 ± 0.55	0.18 ± 0.46	0.18 ± 0.39		0.14 ± 0.35	0.13 ± 0.40	0.13 ± 0.22
	Processed meat	1.62 ± 1.91	1.75 ± 1.94	1.57 ± 1.75		1.6 ± 1.86	1.51 ± 1.79	1.51 ± 1.6
	Tuna	0.34 ± 0.55	0.34 ± 0.58	0.34 ± 0.61		0.38 ± 0.62	0.34 ± 0.53	0.35 ± 0.58
	Fish	2.29 ± 2.04	2.01 ± 1.69	1.88 ± 1.51		2.16 ± 1.85	2.06 ± 1.74	2.09 ± 1.93
	Small fish	0.68 ± 1.16	0.62 ± 1.04	0.64 ± 1.3		0.65 ± 1.08	0.68 ± 1.23	0.71 ± 1.27
	Roe	0.51 ± 0.96	0.45 ± 0.78	0.39 ± 0.6		0.42 ± 0.75	0.4 ± 0.96	0.35 ± 0.62
	Squid and octopus	0.95 ± 1.17	0.86 ± 0.89	0.83 ± 0.77		0.83 ± 0.94	0.84 ± 1.11	0.79 ± 0.78

	Shellfish	0.59 ± 0.91	0.52 ± 0.68	0.53 ± 0.69		0.44 ± 0.60	0.43 ± 0.71	0.40 ± 0.49
	Processed product	0.86 ± 1.20	0.86 ± 1.17	0.86 ± 1.12		0.83 ± 1.18	0.80 ± 1.04	0.87 ± 1.21
	Egg	4.52 ± 3.43	4.65 ± 3.44	4.30 ± 3.18		5.40 ± 3.92	5.32 ± 3.97	5.11 ± 3.78
	Natto and soy	2.24 ± 2.52	2.13 ± 2.39	2.08 ± 2.39		2.33 ± 2.52	2.13 ± 2.29	2.03 ± 2.25
	Tofu	2.04 ± 2.28	1.97 ± 2.18	2.06 ± 2.41		2.34 ± 2.37	2.31 ± 2.28	2.32 ± 2.53
	Ganmodoki	0.56 ± 0.83	0.57 ± 1.01	0.60 ± 0.84		0.68 ± 1.00	0.66 ± 1.04	0.62 ± 0.85
	Miso soup	3.72 ± 3.49	3.59 ± 3.33	3.38 ± 3.11		3.17 ± 3.03	3.21 ± 3.08	3.15 ± 2.91
	Potato	1.05 ± 1.24	1.14 ± 1.41	1.11 ± 1.27		1.41 ± 1.53	1.41 ± 1.46	1.33 ± 1.32
	Carrot	3.02 ± 3.16	3.05 ± 3.15	3.27 ± 3.46		4.07 ± 4.18	4.09 ± 4.09	4.19 ± 4.38
	Pumpkin	0.98 ± 1.44	1.09 ± 1.56	1.09 ± 1.77		1.12 ± 1.71	1.24 ± 1.76	1.25 ± 2.01
	Other light-colored vegetable	3.34 ± 2.58	3.32 ± 2.62	3.48 ± 3.15		4.53 ± 3.29	4.45 ± 3.15	4.48 ± 3.18
	Green vegetable	1.23 ± 1.59	1.31 ± 1.69	1.25 ± 1.72		1.38 ± 1.80	1.43 ± 1.88	1.34 ± 1.62
	Green leafy vegetable	2.02 ± 2.16	1.97 ± 2.17	2.08 ± 2.35		2.43 ± 2.52	2.56 ± 2.73	2.52 ± 2.62
	Cabbage	2.59 ± 2.33	2.59 ± 2.26	2.53 ± 2.49		2.67 ± 2.48	2.70 ± 2.39	2.59 ± 2.48

	Other green vegetable	1.59 ± 1.79	1.59 ± 1.81	1.53 ± 1.80		2.00 ± 2.13	1.99 ± 2.10	2.02 ± 2.22
	Radish	1.44 ± 1.51	1.47 ± 1.62	1.49 ± 1.69		1.61 ± 1.78	1.59 ± 1.74	1.62 ± 1.73
	Pickled radish	0.80 ± 1.49	0.80 ± 1.43	0.85 ± 1.82		0.61 ± 1.34	0.59 ± 1.39	0.77 ± 2.17
	Burdock bamboo	0.79 ± 1.05	0.85 ± 1.19	0.75 ± 1.04		0.84 ± 1.12	0.84 ± 1.10	0.79 ± 1.38
	Mushroom	1.51 ± 1.63	1.50 ± 1.65	1.45 ± 1.63		2.44 ± 2.28	2.33 ± 2.09	2.23 ± 2.08
	Citrus	0.89 ± 1.40	1.02 ± 1.62	1.19 ± 1.85		1.23 ± 1.92	1.29 ± 1.95	1.31 ± 2.06
	Other fruit	1.09 ± 1.79	1.22 ± 1.84	1.44 ± 2.07		1.66 ± 2.30	1.81 ± 2.36	1.70 ± 2.13
	Butter	0.38 ± 1.11	0.48 ± 1.28	0.50 ± 1.30		0.45 ± 1.22	0.62 ± 1.57	0.66 ± 1.63
	Margarine	0.75 ± 1.68	0.95 ± 1.90	1.17 ± 2.04		0.88 ± 1.80	1.06 ± 2.08	0.96 ± 2.12
	Mayonnaise	1.43 ± 1.78	1.47 ± 1.82	1.33 ± 1.66		1.50 ± 1.76	1.38 ± 1.69	1.27 ± 1.85
	Japanese sweet	1.07 ± 1.93	1.39 ± 2.24	1.64 ± 2.30		1.59 ± 2.68	1.92 ± 2.86	2.20 ± 3.54
	Confectionery	0.64 ± 0.95	0.86 ± 1.13	0.89 ± 0.98		1.01 ± 1.59	1.09 ± 1.52	1.30 ± 1.89
	Yogurt	2.35 ± 3.11	2.66 ± 3.17	2.45 ± 2.80		2.87 ± 3.12	3.19 ± 3.21	3.25 ± 3.35
	Peanuts and almond	0.83 ± 1.68	0.93 ± 1.97	0.99 ± 2.21		1.04 ± 2.24	1.11 ± 2.29	1.05 ± 2.18

	Seaweed	1.30 ± 1.67	1.31 ± 1.72	1.30 ± 1.68		1.52 ± 1.93	1.54 ± 1.92	1.50 ± 1.82
	Deep fried food	2.00 ± 1.78	2.11 ± 1.95	1.99 ± 1.57		1.48 ± 1.43	1.55 ± 1.64	1.44 ± 1.28
	Stir fried food	2.65 ± 2.08	2.66 ± 2.02	2.58 ± 1.74		3.17 ± 2.33	3.08 ± 2.35	2.97 ± 1.90
[a] Genotype of the rs671 polymorphysm in *ALDH2* gene.								
[b] Mean ± SD values of measured participants.								

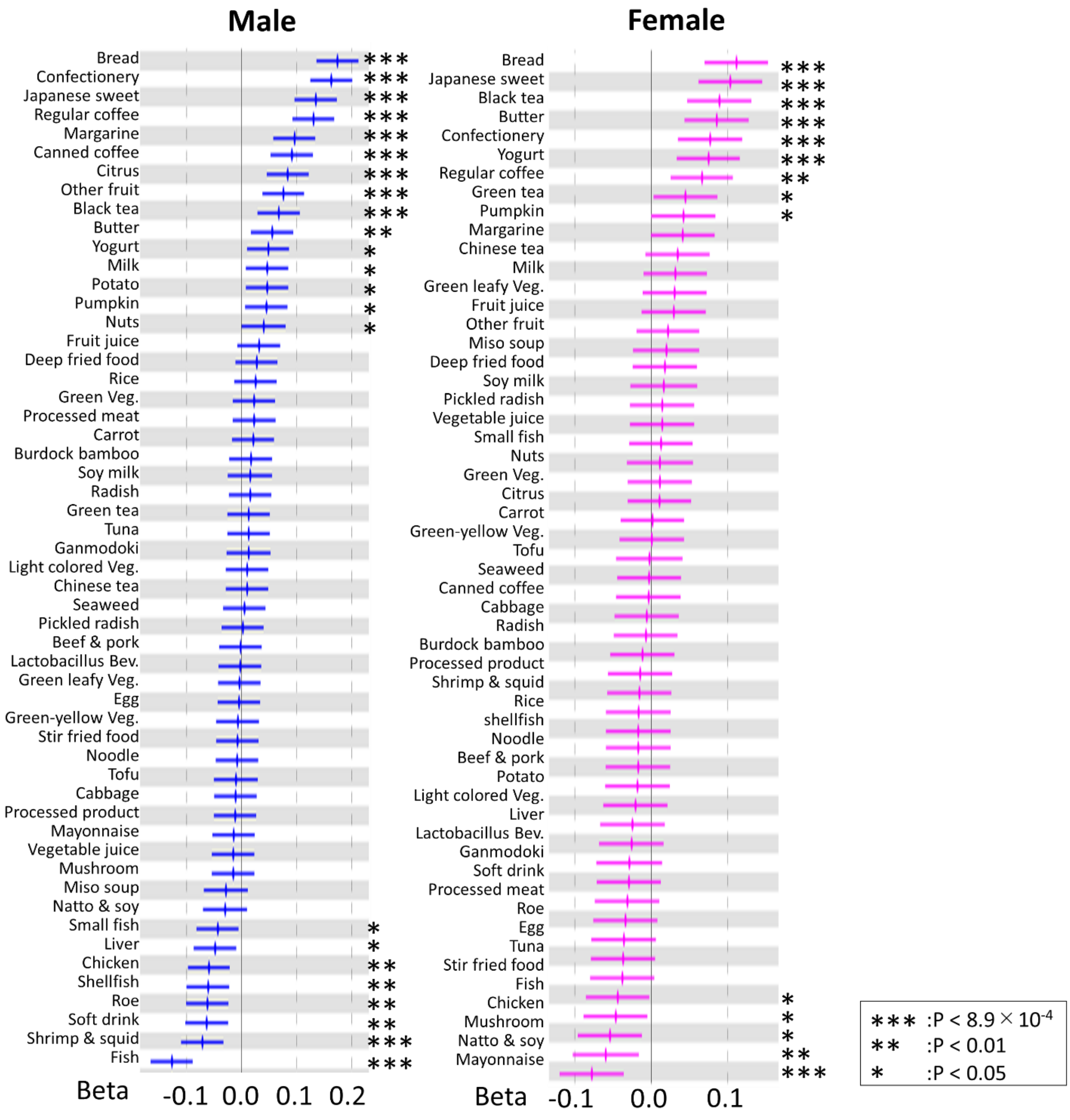

Supplementary Figure S1. Forest plot showing association between DBs and the rs671 polymorphism. The X-axis and Y-axis show standardized beta coefficient ± standard deviation and each of DB, respectively. Spots of blue and pink indicate the results in males and females, respectively. Alc. Cons, Alcohol consumption; Alc. Freq., Alcohol frequency; Lactobacillus Bev., Lactobacillus beverage; Green Veg, Green vegetable; Green leafy Veg., Green leafy vegetable; Green-yellow Veg, Green-yellow vegetable; Light-colored Veg., Light colored vegetable. Asterisks indicate statistical significance (***, $p < 8.9 \times 10^{-4}$; **, $p < 0.001$; *, $p < 0.05$.).

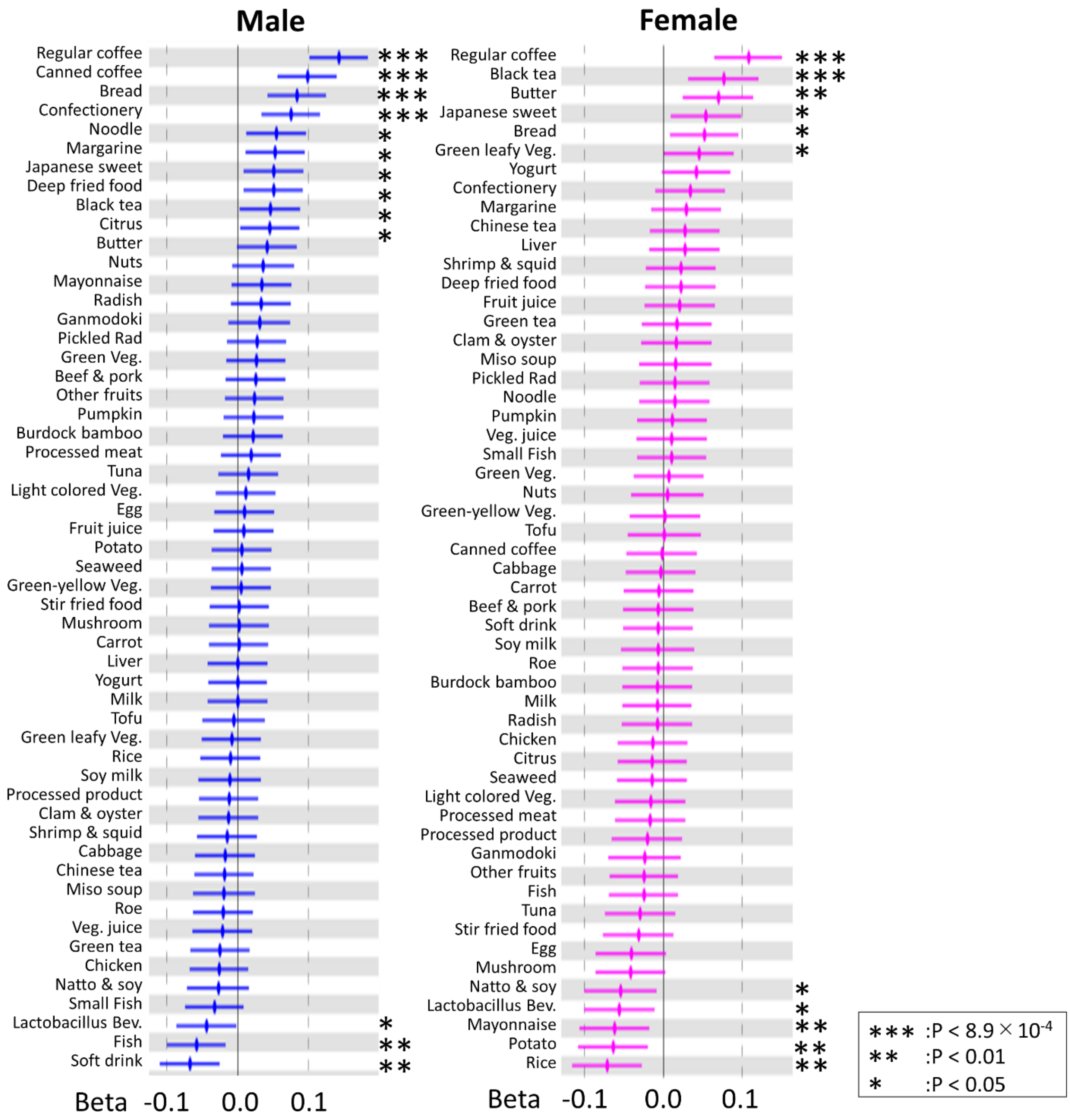

Supplementary Figure S2. Forest plot showing association between DBs and the rs671 polymorphism when adjustment with alcohol consumption and alcohol frequency. The X-axis and Y-axis show standardized beta coefficient ± standard deviation and each of DB, respectively. Spots of blue and pink indicate the results in males and females, respectively. Alc. Cons, Alcohol consumption; Alc. Freq., Alcohol frequency; Lactobacillus Bev., Lactobacillus beverage; Green Veg, Green vegetable; Green leafy Veg., Green leafy vegetable; Green-yellow Veg, Green-yellow vegetable; Light-colored Veg., Light colored vegetable. Asterisks indicate statistical significance (***, $p < 8.9 \times 10^{-4}$; **, $p < 0.001$; *, $p < 0.05$.).
